# Comparison of Salivary and Serum Alkaline Phosphates Level and Lactate Dehydrogenase Levels in Patients with Tobacco Related Oral Lesions with Healthy Subjects - A Step Towards Early Diagnosis

**DOI:** 10.31557/APJCP.2020.21.4.983

**Published:** 2020-04

**Authors:** Gaurav Goyal

**Affiliations:** *Departmen of Oral Medicine and Radiology, Genesis Institute of Dental Sciences and Research, Ferozepur, Punjab, India. *

**Keywords:** Salivary alkaline phosphates, salivary lactate dehydrogenase, squamous cell carcinoma, early diagnosis

## Abstract

**Aim:**

To evaluate and compare salivary and serum levels of Alkaline Phosphates and Lactate Dehydrogenase in patients without the habit of tobacco, in patients with the habit of tobacco, in patients with benign oral lesions and in patients with oral premalignant lesions and oral malignant lesions.

**Material and Methodology:**

This study was comprised of 500 subjects, Group I: 100 healthy individuals without the habit of tobacco usage formed the control group. Group II: 100 patients with the habit of tobacco/ smoking consumption without any oral lesion. Group III: 100 patients with benign oral lesions. Group IV: 100 patients having the history of tobacco consumption and having apparent precancerous lesions like leukoplakia, erythroplakia. Group V:100 patients having frank oral cancer. The grade of dysplasia in these patients was statically correlated with the levels of serum and salivary ALP and LDH.

**Results:**

This study revealed that there was high expression of both serum and salivary ALP and LDH in group IV and Group V as compared with the other groups and mean difference showed a statistically significant p value of less than 0.01. This study revealed that the in group V, the highest level of serum and salivary ALP was found in those patients who were reported with poorly differentiated oral cancer.

**Conclusion:**

Both Alkaline phosphates and Lactate dehydrogenase could be considered a sensitive markers for the detection of dysplasia with already existing precancancerous and cancerous lesions.

## Introduction

Cancer is the second most common disease in India responsible for maximum mortality with about 0.3 millions deaths per year (Imran et al., 2012; Dhivyalakshmi and Uma Maheswari, 2014). In India the incidence of oral cancer is about 3-7 times more common as compared to resource rich countries (Dhivyalakshmi and Uma Maheswari, 2014; Misra et al., 2009). India tops in the prevalence of oral cancer in the world and remains the commonest cancer amongst the male population. Oral cancer is the third most common cancer in India after cervical and breast cancer amongst women (Khan, 2012). In India, the age standardized incidence rate of oral cancer is reported at 12.6 per 100,000 people (Khan, 2012). The increased prevalence of the oral cancer in the Indian subcontinent seems to be due to the high exposure to sunlight due to farming, smoking and other smokeless tobacco habits, alcohol, spicy food, and neglect of overall oral health. It is said that one third of all oral cancers are preventable and one third of them occur due to risk factors (Khan, 2012). The highest age-adjusted incidence for oral cancer is highest in India, i.e. 15.7 per100, 000 and lowest in Japan which is 0.2 per 100,000 and the difference is predominantly due to use of tobacco between the two nations. In the West, the cancer of tongue and floor of mouth is common whereas in Indian subcontinent the cancers of gingival and buccal mucosa are common due to placement of tobacco quid in the oral cavity. This cancer of gingivobuccal complex is termed as Indian oral cancer (Oral Cancer Prevention and Research Foundation, India) (Khan, 2012).

Oral squamous cell carcinoma (OSCC) or Oral Cancer is the sixth most common human cancer that encompasses at least 90% of all oral malignancies. OSCC is recognized to have 50%, five year survival rate( Massano et al., 2006; Priya Shirish et al., 2012). The most common precancerous lesions are oral leukoplakia (OL) and oral erythroplakia. If precancerous lesions are detected and treated early then the conversion to cancer is averted ( Massano et al., 2006; Priya Shirish et al., 2012). Despite the advances made in the therapeutic modalities via multidisciplinary approaches, survival rate for OSCC has not significantly improved. This motivates the search of factors which will help in the early diagnosis and management. Early diagnosis and prompt treatment will avert mutilating surgery, improve patient’s quality of life and can decrease morbidity and mortality associated with cancer.

Recently, the role of tumor markers in management of head and neck cancer has received increasing attention. Tumor markers in serum, tissue and other body fluids during neoplastic process are of clinical value in the management of patients with various body cancers (Priya Shirish et al., 2012; Denny and CM, 2005). Collecting blood for investigation is an invasive procedure and has a potential risk of disease transmission through needle stick injuries (Priya Shirish et al., 2012; Denny and CM, 2005). Despite the absence of charisma, however, a growing number of researchers are finding that saliva provides an easily available, non-invasive diagnostic medium for rapidly widening range of disease and clinical situations. Saliva based diagnostics are more attractive as they are more accessible, accurate, less expensive and presents less risk of infection to the patient, health care worker and cross infection. With all these above mentioned added advantages saliva can serve as diagnostic tool as compared to serum. 

Cellular Alkaline phosphatase (ALP) is increasingly recognized as an important marker of induction of tumor cell differentiation (Dhivyalakshmi and Uma, 2014; Xiao et al., 2007). Cancer patients with bone metastasis mainly had elevated values of bone isoform ALP. Elevated ALP was due to bone destruction and compensation of osteoblastic activity mediated by an increased production of inflammatory mediators such as TNF-alpha (Priya Shirish et al., 2012). The enzyme lactate dehydrogenase (LDH) is widely found in almost all cells of body tissues. Basically LDH enzyme is mainly concentrated in the heart, liver, red blood cells, kidneys, muscles, brain, and lungs. Increased activity of serum LDH is considered to be sign of cellular necrosis (Priya Shirish et al., 2012; Bigler et al., 2009) So, Serum LDH levels have been used as a biochemical marker in diagnosis in various cancers like oral, laryngeal and breast cancer. Increased LDH levels are due to increased mitotic index and more lactic acid production by tumor cells due to breakdown of glycoprotein (Priya Shirish et al., 2012; Bigler et al., 2009). As the magnitude of dysplastic changes increase in leukoplakia it is logical to expect increase in values of LDH. The profile of salivary LDH is similar to that found in oral epithelium, indicating that the major source of salivary LDH is probably the oral epithelium-shedding cells (Nagler et al., 2001).

Carcinogenic changes have tremendous influence in increasing LDH activity. These carcinogenic changes may lead to decreased lactate to pyruvate conversion resulting in anomaly in the regeneration of nicotinamide adenine dinucleotide (NAD+) which may interfere with glycolysis part of carbohydrate metabolism (Kavyashree et al., 2016; Joshi and Golgire, 2014). The development of cancer is associated with a high glycolytic activity with a shift from aerobic respiration to anaerobic glycolysis. Malignant tumor tissue or contiguous tissue damaged by tumor liberates enzymes into circulation which contributes towards abnormal increase in enzyme levels (Joshi and Golgire, 2014; Priya Shirish et al., 2012).

The LDH in the whole saliva within the oral cavity originates from various sources as it is a combination of secretions from both major and minor salivary glands, diffusion of fluids through the oral epithelium and gingiva, material originating from gastrointestinal reflux, and cellular and other debris. Some studies found out that whole saliva LDH is nonglandular in origin and probably oral epithelium is the major source for this nonglandular LDH (Misra et al., 2009; Priya Shirish et al., 2012; Imran et al., 2011). It might be because of the pathological alternations of oral epithelium such as dysplasia or cancer which may result in alternation of LDH levels in the saliva. Therefore, salivary LDH may be analyzed for possible oral mucosal pathologies (Kavyashree et al., 2014; Priya Shirish et al., 2012). The similarity between the profile of LDH in whole saliva and the oral epithelium supports the hypothesis that salivary LDH is predominantly of extra glandular origin (Priya Shirish et al., 2012; Lingen et al., 2008). Consequently, LDH concentration in saliva, as an expression of cellular necrosis, could be a specific indicator for oral lesions that affect the integrity of the oral mucosa (Priya Shirish et al., 2012; Lingen et al., 2008). Therefore salivary LDH and ALP levels may be evaluated for possible oral mucosal pathologies. So, the aim and objective of the study was to evaluate weather salivary analysis of Alkaline Phosphates (ALP) and Lactate dehydrogenase (LDH) can be used for early diagnosis of Oral Cancer. Furthermore, to assess the levels of salivary LDH and ALP isoenzymes and histopathologic grades of dysplasia in precancerous/ cancerous lesions.

## Materials and Methods

Ethical clearance was obtained from the institutional ethical board before starting the study. Written informed consent of the patient was obtained.

The study was comprised of 500 subjects reporting to the Department of Oral Medicine and Radiology. These patients was further subdivided into four groups of matched age and gender.

Group I: 100 healthy individuals without the habit of tobacco usage formed the Control group. 

Group II: 100 patients with the habit of tobacco/ smoking consumption without any oral lesion.

**Figure 1:**
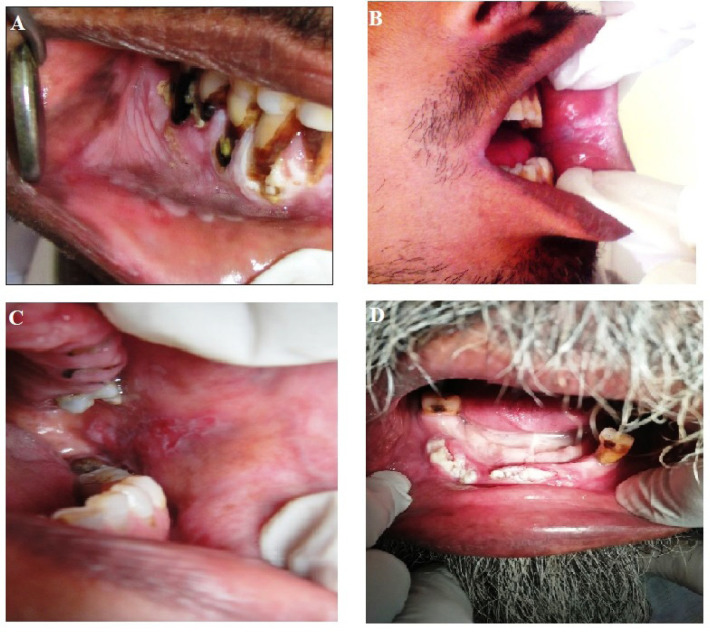
A, Benign oral lesions such as Tobacco pouch keratosis, Stomatitis Nicotina; B and C, Precancerous lesions like leukoplakia, erythroplakia, Lichen planus; D, Frank Oral Cancer

Group III: 100 patients with benign oral lesions such as Tobacco pouch keratosis, Stomatitis Nicotina etc (Fig 1A)

Group IV: 100 patients having the history of tobacco consumption and having apparent precancerous lesions like leukoplakia, erythroplakia, Lichen planus etc (Fig 1 B and C).

Group V: 100 patients with frank Oral Cancer (Fig 1 D).

*Exclusion Criteria*

Patients suffering from systemic conditions like cardiovascular disease, anemia, liver or heapatobilliary diseases, kidney diseases, Bone diseases, pancreatic diseases, blood dyscrasis, stroke, muscular dystrophy, diabetes, drugs like anesthetics, narcotics, aspirin, postmenopausal women, patients with other mucosal lesions and the local and systemic conditions which increased the level of LDH and ALP was excluded from the study (Priya Shirish et al., 2012; Dhivyalakshmi et al., 2014). In all the groups, salivary levels of LDH and ALP was assessed and was correlated with serum LDH and ALP levels. Further patients having precancerous lesions and frank oral cancer, an effort was made to correlate salivary and serum LDH and ALP levels with the grade of dysplasia.

Patients having precancerous lesions or frank oral cancer was subjected to histopathological examination after selecting an appropriate site of biopsy (Toludine blue staining). The grade of dysplasia in these patients was statically correlated with the levels of serum and salivary ALP and LDH. 

Saliva Collection: 5 ml of unstimulated saliva was collected from each of the patients by the spit method in a calibrated test tube. Care was taken to see that the volunteers did not consume food or chew gum at least one hour before the collection procedure. Following collection, saliva was immediately centrifuged at 2,500 rpm for 15 minutes to remove squamous cells and cell debris. The resulting supernatant was separated into 1 ml aliquots and subjected for further biochemical assay analysis using standard kit method. The LDH and ALP concentrations was expressed in terms of IU/L. 

Serum Collection: The subject was made to sit comfortably in a chair with the arm extending straight from the shoulder. The anticubital fossa was exposed and a tourniquet was applied 1.5-2 inch above the anticubital fossa. The area was rendered aseptic with a sponge soaked in 70% alcohol. Using a 5ml sterile, disposable syringe and a 24 gauge needle, the anticubital vein was punctured and 2.5 ml blood was drawn. The tourniquet was then relieved and needle removed, simultaneously applying alcohol soaked cotton on the puncture site. The collected blood was transferred to sterile blood collecting tube. This was centrifuged at 2,000 rpm for five minutes and the serum was separated. Then this further subjected for estimation of LDH and ALP levels.

*Biopsy procedure*

Suspected precancerous/cancerous lesions was subjected to Toludine Blue staining to note the most appropriate site for biopsy. 1% acetic acid was applied on the site having lesion followed by application of 1% toludine blue staining. After 30 seconds again 1% acetic acid was applied to neutralize all the blue dye except where it had been taken up by dysplastic tissue. The area which retains the maximum amount of dye was selected as a primary site for incisional biopsy. Under aseptic conditions and local anesthesia adequate amount of tissue was incised from the selected site. The biopsy tissue was subjected to histo-pathological examination to check for presence of any dysplasia. Dysplasia if present was further categorized into mild dysplasia, moderate dysplasia, severe dysplasia, Carcinoma in situ and Squamous Cell Carcinoma.

*Statistical Analysis*

Statistically significant differences between groups were compared using analysis of variance (ANOVA) and Duncan test. The relationship between several quantitative variables in the groups was measured using Pearson correlation coefficient and significance test performed. The data was displayed as means ± standard deviation (SD). Sensitivity-specificity analysis was done and results expressed in likelihood ratio (LR). The correlation of the histological tumor differentiation with the serum LDH and ALP levels was done using Spearman’s correlation. The level of significance was set at P < 0.01.

## Results

**Figure 2:**
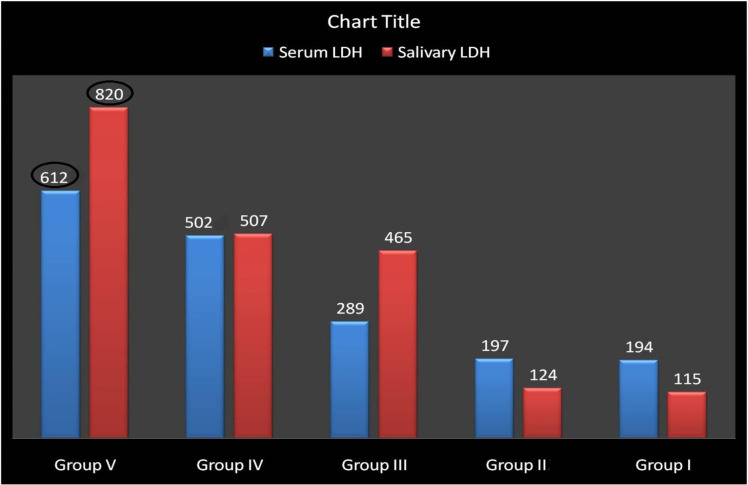
Bar Diagram Showing the Correlation of Serum and Salivary LDH between the Groups

Correlation of Serum LDH between the Groups Patients in group V (frank oral cancer) having the value between 599 to 878 U/L (Mean = 612 U/L). Patients in the Group IV (Precancerous lesions) having the value between 344 to 612 U/L (Mean = 502 U/L). Patients in the Group III (Benign Lesions) was having the value between the 236 to 378 U/L (Mean= 289 U/L). Patients who used tobacco, but do not manifest with a lesion, showed Serum LDH levels with a range from 145 to 235 U/L (Group II) Mean = 197 U/L. Patients who did not use tobacco and who did not present with a lesion on intraoral examination had Serum LDH levels ranging from 155 to 205 U/L (Group I- Control Group) Mean= 194 U/L ( Figure 2).

*Correlation of Salivary LDH between the Groups*

Patients in group V (Oral Cancer) patients was having the value between 599 to 1100 U/L (Mean = 820 U/L). Patients in the Group IV (Precancerous lesions) having the value between 412 to 917 U/L (Mean = 507 U/L) and Patients in the Group III (Benign Lesions) having the value between the 147 to 509 U/L (Mean= 465U/L). Patients who used tobacco, but do not manifest with a lesion, showed salivary LDH levels with a range from 93 to 298 U/L (Group II) Mean = 124 U/L. Patients who did not use tobacco and who did not present with a lesion on intraoral examination had Salivary LDH levels ranging from 80 to 257 U/L (Group I- Control Group) Mean= 115U/L (Figure 2).

**Figure 3:**
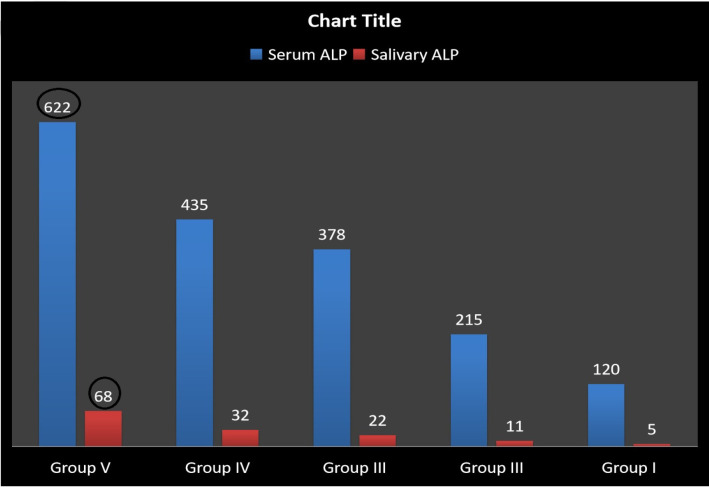
Bar Diagram Showing the Correlation of Serum and Salivary ALP between the Ggroups

Correlation of Serum ALP between the Groups The mean Serum ALP value (Group V) of patients with habit and oral cancer is 622 IU/L. The mean Serum ALP value (Group IV) of patients with habit and oral cancer is U/L and with the precancerous lesion is 435 IU/L. The mean Serum ALP value of patients with habit and the benign lesion (Group III) is 387 IU/L. The mean Serum ALP value for patients with habit but without the presence of a lesion (Group II) is 215 IU/L.The mean Serum ALP value for patients without a habit or lesion (Group I) is 120 IU/L. The patients having the habit of using tobacco with precancerous and Cancerous lesions having higher Serum ALP values compared to the other three groups (Figure 3). Correlation of Salivary ALP between the Groups The mean Salivary ALP value (Group V) oral cancer is 68 IU/l. The mean Salivary ALP value (Group IV) of patients with habit and with the precancerous lesion is 32 IU/L. The mean Salivary ALP value of patients with habit and the benign lesion (Group III) is 22 IU/L. The mean Salivary ALP value for patients with habit but without the presence of a lesion (Group II) is 11 IU/L. The mean Salivary ALP value for patients without a habit or lesion (Group I) is 5 IU/L. The patients having the habit of using tobacco with precancerous and Cancerous lesions having higher Salivary ALP values compared to the other three groups (Figure 3).

**Figure 4:**
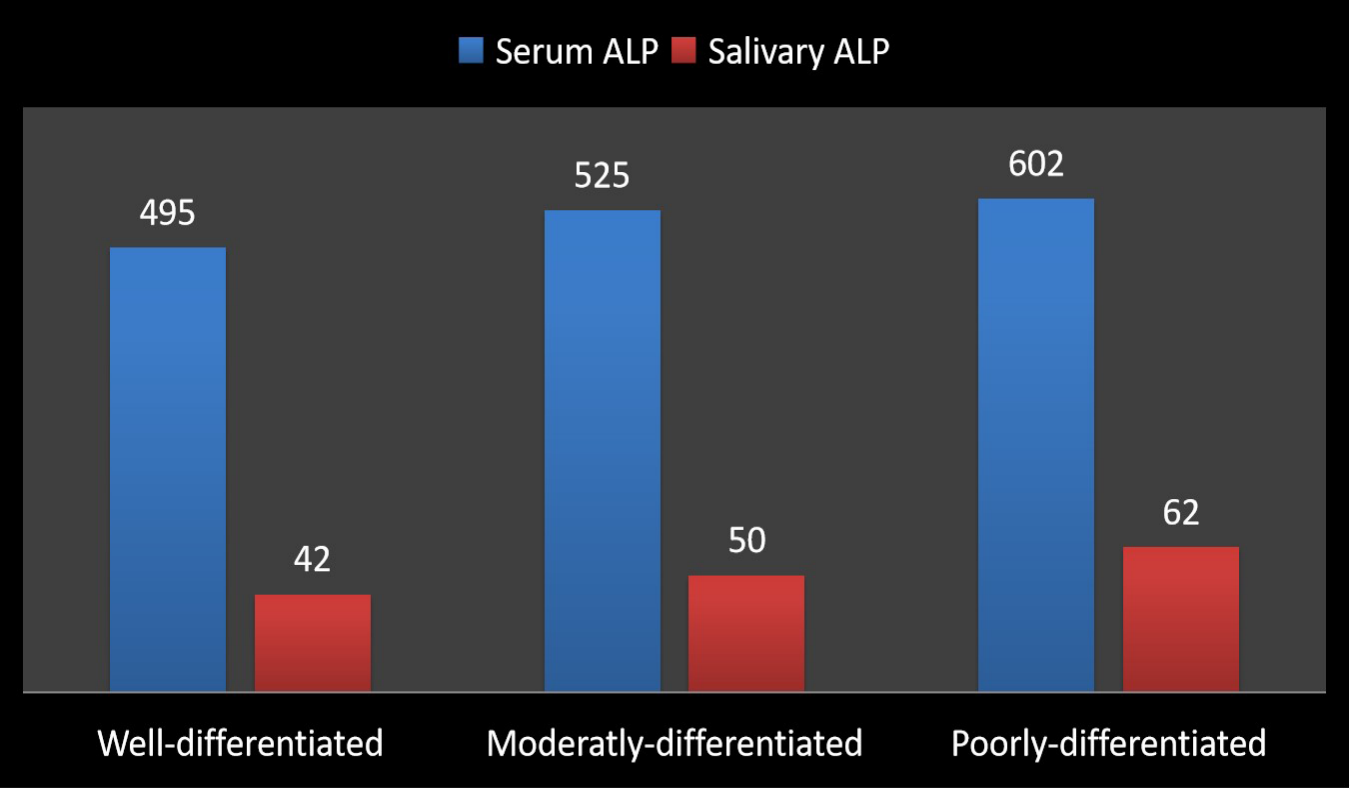
Bar Diagram Showing the Correlation of Salivary and Serum ALP with Histo-Pathologic Grade of Dysplasia in Group V

**Figure 5:**
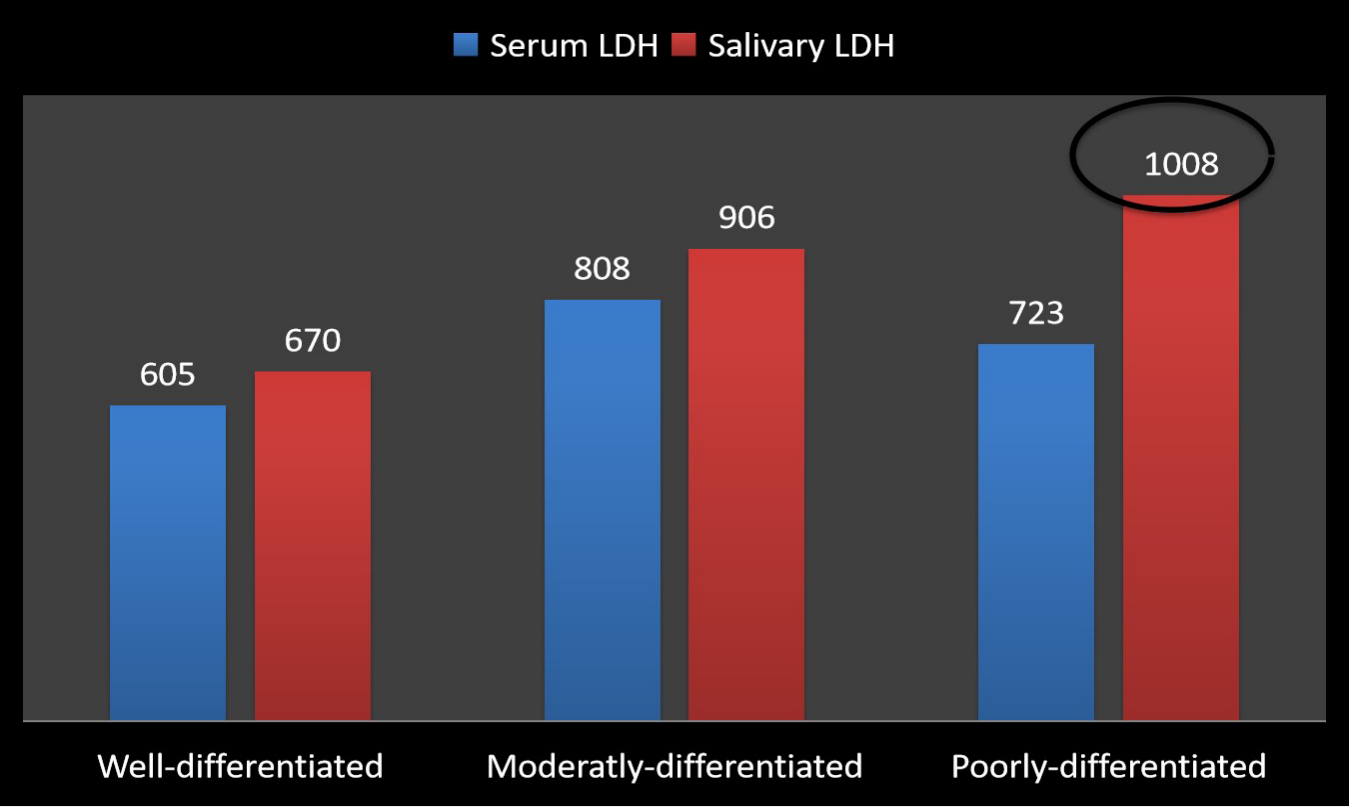
Bar Diagram Showing Correlation of Salivary and Serum LDH with Histo-Pathologic Grade of Dysplasia in Group V

Correlation of Group V with Histo-pathologic Grade of Dysplasia This study revealed that the in group V, the highest level of serum and salivary ALP was found in those patients who were reported with poorly differentiated oral cancer. The cases of well differentiated oral cancer have a significantly less level of serum and salivary ALP levels as compared to the cases of moderately and poor differentiated Squamous cell carcinoma (Figure 4). This study also revealed the level of salivary LDH was significantly on the higher side in poorly differentiated compared to moderately and poor differentiated Squamous cell carcinoma. However, the mean highest serum level of LDH was found to be in moderaltly differentiated Squamous cell carcinoma (Figure 5). The reason could be that the high rate of glucose utilization found in more poorly differentiated tumors and has a higher component of aerobic oxidative metabolism and a relatively lower contribution from anaerobic metabolism found in poorly differentiated tumors leading to lower LDH levels. So, this study clearly indicated that the level of serum and salivary ALP and salivary LDH were correlated with histo-pathologic Grade of Dysplasia.

*Correlation of Group IV with histo-pathologic grade of dysplasia*

**Figure 6.**
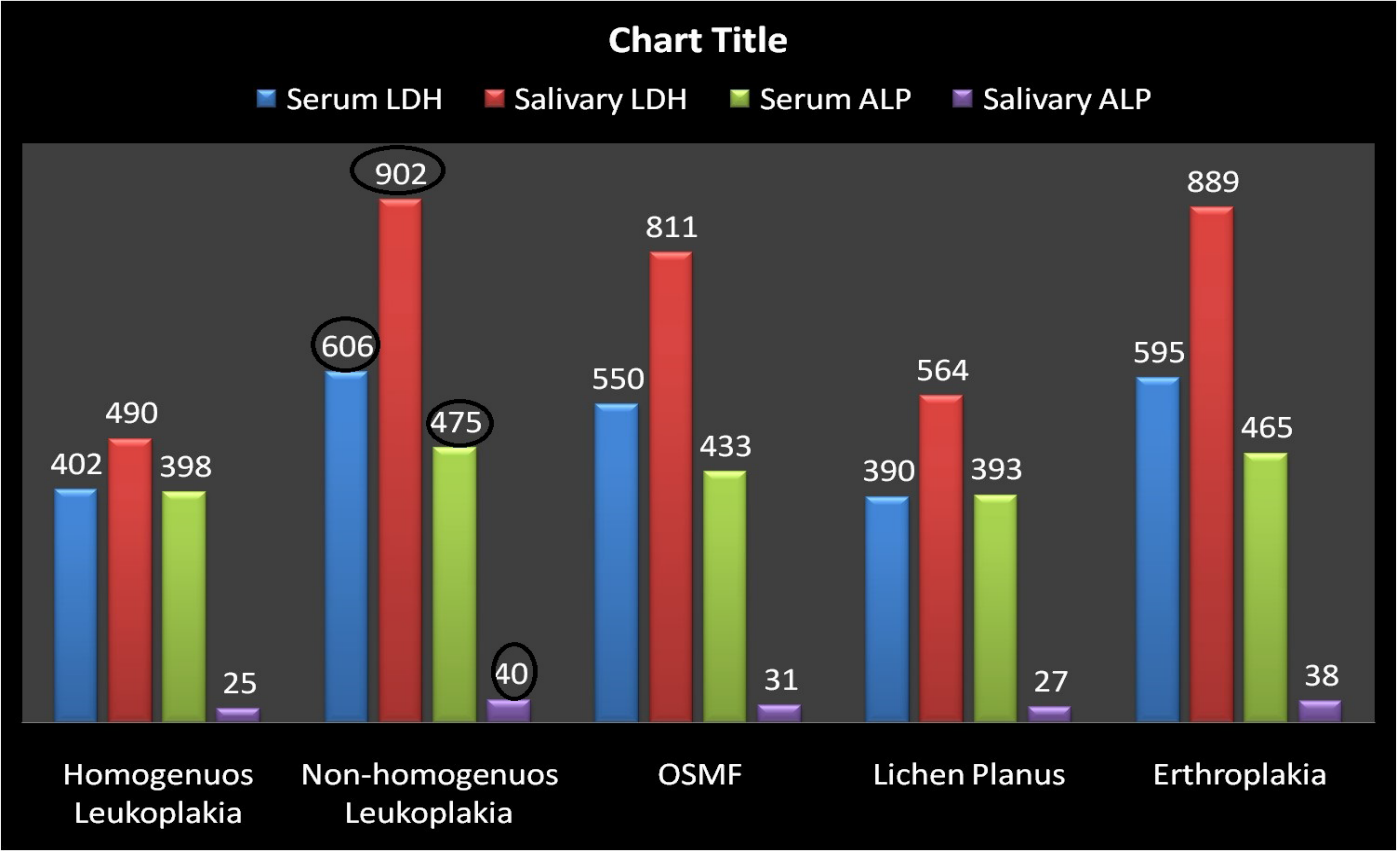
Correlation of Salivary and Serum ALP and LDH with Histo-Pathologic Grade of Dysplasia in Group IV

The group IV patients had been further divided according the histo-pathological grade of dysplasia such as mild dysplasia, moderate dysplasia, and severe dysplasia. The mild and moderate dysplasia had been encountered in homogenous leukoplakia, lichen planus and OSMF. Severe dysplasia had been reveled in cases of non homogeneous leukoplakia as well as Erthyroplakia. The level of serum and salivary ALP and LDH had been assessed in all the cases and results of the study showed that the highest level of serum and salivary ALP and LDH had been noticed in cases of homogeneous leukoplakia and erthyroplakia (Figure 6). This clearly indicated that when the level of dysplasia raised, there was increased level of serum and salivary ALP and LDH. So, if the patients had high level of serum and salivary ALP and LDH, we could expect the more chances of conversion of precancerous lesions into the malignant cancerous lesions. 

*The analysis of sensitivity and specificity in serum and salivary LDH and ALP*

The sensitivity and specificity levels have been assessed by F test. The study revealed that occurrence of squamous cell carcinoma was high in those cases in which the level of serum and salivary ALP 500 and 50 IU/L respectively and further reveled LR>1, sensitivity 83%, specificity 79% in serum ALP and LR>1 and sensitivity 80%, specificity 73% in salivary ALP with significant p value of less than 0.01. We also observed the similar findings in serum and salivary LDH with LR>1, sensitivity 93%, specificity 89% in serum LDH and LR>1, sensitivity 87%, specificity 82% in salivary LDH with significant p value of less than 1 (Table 1).

**Table 1:** Correlation of Sensitivity, Specificity, Positive Predictive Value and Negative Predictive Values among the Groups

	Serum ALP	95%CI	Salivary ALP	95%CI	Serum LDH	95%Ci	Salivary LDH	95%CI
Sensitivity	0.91	0.81-0.97	0.8	0.70-0.88	0.93	0.81-0.98	0.87	0.74-0.96
Specificity	0.79	0.71-0.90	0.73	0.68-0.87	0.87	0.72-0.93	0.83	0.71-0.89
Positive predictive value	0.2	0.1-0.5	0.3	0.1-0.6	0.4	0.1-0.7	0.5	0.1-0.7
Negative predictive value	0.5	0.2-0.7	0.5	0.2-0.7	0.6	0.2-0.8	0.5	0.1-0.7
Likelihood ratio								
Two tailed *P*-value	<0.01		<0.01		<0.01		<0.01	

## Discussion

Globally, SCC is the sixth most common cancer with an annual incidence of over 300,000 cases, out of which 62% arise in the developing countries (Imran et al., 2011; Parul et al., 2017). India tops in the prevalence of oral cancer in the world and remains the commonest cancer amongst the male population. In comparison with the U.S. population, where oral cancer represents only about 3% of malignancies, it accounts for over 30% of all cancers in India. 4 in 10 of all cancers are oral cancers in India (Parul et al., 2017). Such data make the oral cancer an important public health matter which is responsible for 3% to 10% of cancer mortality worldwide (Parul et al., 2017). The risk of developing precancerous lesions like leukoplakia, oral malignancies and other oral mucosal lesions is substantially increased by tobacco smoking or the use of smokeless tobacco. The risk of developing oral cancer depends on the duration and frequency of tobacco usage. Both of these substances are associated with an increased oral cancer risks of 50-folds over that of nonusers. Serum and Salivary bio-markers proved to be a great signs of the development of the cancers in all the regions of body. However, very less studies with significant findings revealed the role salivary bio-markers in squamous cell carcinoma. All the previous conducted studies that have many flaws such as small sample size, no correlation of salivary ALP and LDH, no correlation with histo-pathological grade of dysplasia etc. 

To our knowledge this is first study that assessed the levels of salivary and serum ALP and LDH in various stages of tobacco related oral disease starting from benign stages to precancerous stages and to malignant change. Moreover we also correlation with the various histological grade of dysplasia with levels of salivary and serum ALP and LDH had also been done. 

Many researchers revealed that the ALP is a tumor associated antigen and had been used to assessed the drug efficacy. The association of serum ALP with tumors of liver, lung, bone breast and colorectal had been proved by various authors (Ying et al., 2015; Damera et al., 2011; Sonpavde et al., 2010). In addition to that, some other diseases such as cholestasis, hepatitis, and infiltrative liver disease had also showed increased levels of ALP. However, till date the association of salivary and serum ALP with the pathological grade of dysplasia has not been evaluated till now. Banseria et al., (2014) in a study, to find the relation between ALP levels with grading and staging OSCC observed raised ALP in 8% cancer patients. The mean serum ALP value raised from Stage I-IV cancer and this was found to be statistically significant. In the present investigation, the mean ALP level in patients with advanced stage was significantly higher than with early stage. Similar results were obtained by Swetha et al., (2017) explaining the ectopic production of ALP at tumor site. Aminian et al., (2011) retrospectively examined serum ALP in esophageal carcinoma and found that mean ALP was higher in patients with LNM (141 U/L) than with negative node involvement (116 U/L), with a mean difference of (25 U/L). Thus, elevated ALP levels in patients with esophageal cancer may predict the LNM.This study revealed that there was high expression of both serum and salivary ALP and LDH in group IV and Group V as compared with the other groups and mean difference showed a statistically significanct p value of less than 0.01. The results of this study was in correlation with results of the study conducted by Hariharan et al., (1977) and Pereira et al., (2015). Both studies also assessed that the level of serum LDH and ALP and found significantcorrelation in cancerous group as compared with the control group. One-way ANOVA analysis of serum and salivary LDH and ALP between control, HNSCC revealed an extremely significant difference in serum and salivary ALPand LDH (P <0.01) only between the groups which is in agreement with the findings of Pereira et al., (2015).

Bassalyk et al., (1992) revealed that the serum ALP and LDH increased by 1-5- 6 times in the carcinoma cases as compared with the control group. Some of the studies also revealed that the serum ALP and LDH levels were more in precancerouslesion as compare with the control, however, the mean difference was non significant. In another study, Rassam et al., (1995) pointed out heat-stable ALP as a useful tumor marker in the management of HNC. 

As per the broders classification, the squamous cell carcinoma has been divided into various histological grades. We have found that the levels of salivary and serum ALP and LDH was consistently increasing when the grade of dysplasia was advancing. However, the levels of serum LDH was highest in cases of moderately differentiated carcinoma as compared to poorly differentiated carcinoma. The possible reason could be that the high rate of glucose utilization (indicated by hexokinase activity) found in more poorly differentiated tumors has a higher component of aerobic oxidative metabolism (indicated by malate dehydrogenase activity) and a relatively lower contribution from anaerobic metabolism (indicated by LDH activity) than do the rates found in more differentiated tumors leading to lower LDH levels (Bhattacharjee et al., 2018). Banerjee et al., (1989) also studied the histochemical distribution of lactate, isocitrate and succinate dehydrogenases in normal oral epithelium and in well-differentiated SCC and found it to be more in the malignant cells.

Shpitzer et al., (2009), involving five salivary parameters, elevated salivary LDH levels were observed in tongue cancer patients when compared to healthy controls. Shetty et al., (2012) who also found that total salivary LDH is higher in oral leukoplakia and OSCC as compared to controls. Priya Shirish et al., (2012) who concluded that LDH activity increased in serum as well as saliva in patients with oral leukoplakia and oral squamous cell carcinoma in comparison to normal control.

The sensitivity and specificity of salivary and serum ALPand LDH level was significantly higher for the SCC group showing increased probability for SCC. Other studies have shown that higher serum LDH levels were seen more in metastatic disease especially relapse, liver metastasis, and nodal metastasis. In locoregional disease, the level was rarely >2 times the normal level. For serum ALP levels, results for both groups were not significant.

Limitation: As the level of serum and salivary ALP and LDH has been increased in cases of the Periodontitis and we have all the cases associated with periodontitis. So, it is logical to expect that the increased level of serum and salivary ALP and LDH could be due to Periodontitis. So, there should be longitudinal studies needs to conduct in future to divide the patients of same groups into further subgroups of periodontitis such as mild, moderate and severe periodontitis. 

In conclusion, both Alkaline phosphates and Lactate dehydrogenase could be considered a sensitive markers for the detection of dysplasia with already existing precancancerous and cancerous lesions. Hence, helpful in early detection of oral carcinoma. Salivary LDH and ALP estimation could prove to be a valuable substitute to serum LDH and ALP as a biochemical marker, as it is a simple, non-invasive procedure and easily accepted by the patient. However, the changes in serum are more striking than that is saliva while in a local malignancy like Oral Precancerous and OSCC, the changes could be more prominent in saliva than that in blood. So, we could propose that salivary analysis of LDH and ALP could be used as an efficient, noninvasive and friendly new tool for early diagnosis of oral cancer and monitoring the conversion of precancerous lesion into cancerous lesion. 

*Implications for Future Research*

With a collection of single drop of saliva we could have an insight about the histo-pathological nature of precancerous / cancerous lesions. The levels of salivary ALP and LDH could give us an idea about the amount of dysplasia with precancerous / cancerous lesions. Hence, we can help to predict the future progression and outcome of the patient. 
